# Angular threshold for intraocular pressure elevation during robot-assisted radical prostatectomy in Trendelenburg position

**DOI:** 10.3389/fmed.2026.1769460

**Published:** 2026-05-15

**Authors:** Xinyue Liu, Chen Wang, Yumei Zhang, Chunjing Li, Jingjing An

**Affiliations:** 1 Operating Room, Department of Anesthesiology, West China Hospital, Sichuan University, Chengdu, China; 2 Nanjing Mindray Bio-Medical Electronics Co., Ltd., Nanjing, Jiangsu, China; 3 Operating Room, Department of Anesthesiology, West China Hospital, Sichuan University/West China School of Nursing, Sichuan University, Chengdu, China

**Keywords:** intraocular pressure, predictive model, risk factors, robot-assisted radical prostatectomy, Trendelenburg position

## Abstract

**Introduction:**

This study aimed to determine the angular threshold and key factors influencing intraocular pressure (IOP) elevation in patients undergoing robot-assisted radical prostatectomy (RARP) in the Trendelenburg position, to optimize patient positioning to mitigate ocular risks.

**Methods:**

This prospective study enrolled patients undergoing RARP between July 2021 and June 2022 at West China Hospital, Sichuan University. The patients were positioned at varying Trendelenburg angles (18°–28°) and leg angles. Intraoperative IOP was measured at 11 predefined time points. Receiver operating characteristic (ROC) curve analysis was used to determine the optimal cutoff values for predicting increased IOP. Univariate and multivariate analyses were used to explore the risk factors associated with increased IOP, and a predictive model for increased IOP was developed based on the risk factors.

**Results:**

A total of 223 patients scheduled for RARP were screened, and 135 patients were included for final analysis. The optimal threshold for Trendelenburg angle was 20.5°, with angles ≥20.5° significantly associated with elevated IOP (*p* = 0.001). Patients with diabetes also exhibited higher IOP risk (*p* = 0.028). Based on risk factors including Trendelenburg position angle group, leg segment angle group, mean arterial pressure, and diabetes history, the predictive model for elevated IOP demonstrated an AUC of 0.721 (95% CI: 0.621–0.820), confirming its discriminative capacity.

**Conclusion:**

Steeper Trendelenburg positioning (≥20.5°), leg angles (≥9.5°), and diabetes history are independent risk factors for intraoperative IOP elevation during RARP. These findings could highlight the importance of individualized patient positioning, favoring milder angles in high-risk patients to reduce ocular complications.

## Introduction

Prostate cancer (PCa) is one of the most common cancers among males worldwide, with about 1.5 million new diagnoses globally each year (1). For localized PCa, the 5-year survival rate was relatively high after proper treatment, but once the PCa developed into the metastasis stage, the survival rate dropped significantly (2). Currently, common treatment options for PCa include radiotherapy, chemotherapy, and surgery. For the localized PCa, the surgical method was still the mainstream treatment, and robot-assisted radical prostatectomy (RARP) has become a crucial treatment for PCa, compared to open and laparoscopic approaches. RARP maximizes the advantages of the surgeon’s experience (3). With real-time camera tracking and more flexible instrument tips, this technique offers better control over pelvic floor preservation and urethral anastomosis (4). Notably, while RARP offers significant surgical advantages, its requirement for specific patient positioning introduces unique physiological considerations that intersect with ocular health, particularly regarding intraocular pressure (IOP) regulation.

IOP is regulated by a delicate balance between aqueous humor production and drainage, influenced by both intrinsic ocular mechanisms (5) and extrinsic factors like posture and intra-abdominal pressure (6). Elevated IOP during surgery is a significant clinical concern (7), with population-based studies reporting a global prevalence of 4–7% for ocular hypertension and 3–5% for primary open-angle glaucoma (POAG). Although IOP was influenced by various factors, including age, ethnicity, and metabolic factors, strongly influence IOP epidemiology (8). Prolonged exposure to pressures >24 mmHg may disrupt ocular blood flow, increasing risks of ischemic optic neuropathy or glaucoma. A case report, such as Weber et al. (9), highlights POVL following minimally invasive surgeries, underscoring the need for preventive strategies. Current literature lacks consensus on safe thresholds for Trendelenburg angle, head elevation, and positioning duration, with most studies focusing on isolated parameters rather than their combined effects.

The elevated IOP during surgery was another important issue affecting postoperative recovery. Recently, IOP elevation during RARP in the Trendelenburg position has emerged as a critical concern in urologic and anesthesiology practice (10). One underlying reason was the Trendelenburg position (characterized by a head-down tilt), which is essential for optimal surgical exposure during RARP. This positioning can lead to significant physiological changes, including increased IOP, and pose risks such as postoperative vision loss (POVL) and ocular perfusion impairment (11). Early studies demonstrated that steep Trendelenburg angles (e.g., 30°–45°) cause a rapid and sustained rise in IOP, often exceeding the normal threshold of 21 mmHg. For instance, Blecha et al. (12) reported a time-dependent IOP increase during prolonged 45° Trendelenburg positioning, while Nishikawa et al. (13) found significantly higher IOP at 30° compared to 25°. These changes are exacerbated by pneumoperitoneum, which further reduces venous return and increases central venous pressure. Therefore, understanding the factors influencing IOP elevation during surgery is vital for improving patient safety (14). This study aimed to determine the angular threshold and key factors influencing intraocular pressure (IOP) elevation in patients undergoing RARP in the Trendelenburg position, to optimize patient positioning to mitigate ocular risks.

## Materials and methods

### Study design and participants

Patients scheduled for RARP between July 2021 and June 2022 at West China Hospital, Sichuan University, were invited to participate in this study. The inclusion criteria were age between 18 and 80 years. The exclusion criteria were: (1) Patients with a history of ophthalmic disease or surgery; (2) patients with spinal issues and incompatible with the Trendelenburg position. The study was approved by the Biomedical Ethics Review Committee of West China Hospital, Sichuan University [Approval No. 2021 Review (682)]. Informed consent was obtained from all participants.

### Procedures

The clinical characteristics, including age, ethnicity, body mass index (BMI), mean arterial pressure (MAP), hypertension history, diabetes history, and angle information, were collected from medical records.

Anesthesia was induced using a standardized regimen: propofol 2.5 mg/kg, sufentanil 0.3 μg/kg, and rocuronium 0.6 mg/kg. Anesthesia was maintained with sevoflurane inhalation at a concentration of 2.0–3.0% combined with a continuous intravenous infusion of remifentanil at 0.1–0.2 μg/kg/min, along with intermittent boluses or continuous infusion of atracurium or rocuronium for neuromuscular blockade. Pneumoperitoneum pressure was maintained at 13 mmHg throughout the procedure.

All surgeries were performed by four experienced urologic surgeons. Following anesthesia, nurses adjusted the operating table to a Trendelenburg angle between 18° and 28°, depending on the surgeon’s judgment of surgical needs. In some cases, the anesthesiologist raised the headrest by 5° to 19° based on intraoperative requirements. At this point, the Trendelenburg angle (TREND), head-up angle (BACK), and leg angle (LEG) were recorded. The LEG was defined as the angle of the operating table’s leg section relative to the horizontal plane. A positive value indicates that the legs were elevated above the horizontal plane (hip flexion), while a negative value indicates that the legs were lowered below the horizontal plane (Supplementary Figure S1).

Intraocular pressure was measured at 11 time points (Supplementary Table S1) using a handheld tonometer. Each eye was measured three times, and readings within ±1 mmHg error were averaged to obtain the final IOP for that eye. To prevent cross-infection, each patient was assigned a dedicated IOP probe, which was covered with a sterile cap when not in use. In this study, an IOP >24 mmHg was defined as elevated IOP (15).

### Statistical analysis

Statistical analyses were performed using SPSS (Version 29.0, IBM Corp., NY, United States). Continuous variables conforming to a normal distribution were expressed as mean ± standard deviation (SD) and compared using student *t*-test, while variables not conforming to a normal distribution were described using medians (Q25, Q75) and compared using the rank-sum test. Categorical variables were presented as *n* (percentage) and compared using the chi-square test or Fisher’s exact test. To explore the best cutoff of TREND and LEG for IOP, IOP was defined as the outcome variable, while the TREND angle and LEG angle were used as independent variables. ROC curves were plotted for each variable, and Youden’s index was calculated across different cutoff values to determine the optimal threshold.

For repeated intraoperative IOP measurements, generalized estimating equations (GEE) were employed to account for within-subject correlation, utilizing an exchangeable working correlation matrix and a linear link function. Time-to-event analysis for elevated IOP (defined as the interval from the completion of patient positioning to the first recorded IOP >24 mmHg) was conducted using Kaplan–Meier curves, with group differences assessed by the log-rank test. To develop the predictive model, variables demonstrating statistical significance (*p* < 0.05) in univariate analysis, alongside clinically relevant factors (e.g., MAP), were incorporated into a multivariable binary logistic regression model. Model performance was evaluated using the area under the curve (AUC). In this study, two-sided *p* < 0.05 indicated statistical significance.

### Ethics approval and consent to participate

The study was approved by the Ethics Committee of West China Hospital of Sichuan University [2021 Review (No. 682)]. All participants were informed about the study protocol and provided written informed consent to participate in the study. I confirm that all methods were performed in accordance with the relevant guidelines. All procedures were performed in accordance with the ethical standards laid down in the 1964 Declaration of Helsinki and its later amendments.

## Results

### Basic characteristics

**Figure 1:**
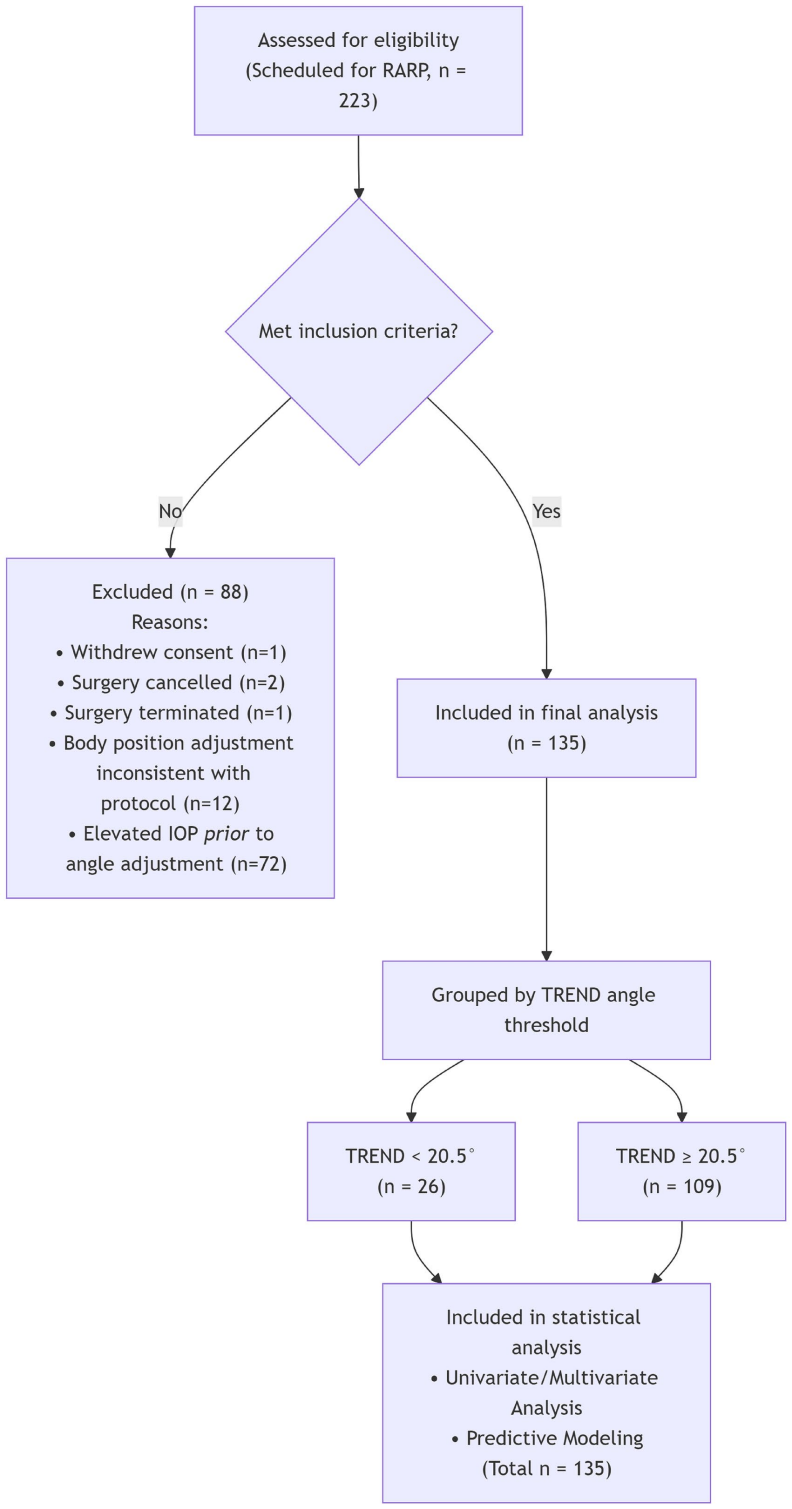
The inclusion and exclusion of participants.

A total of 223 patients scheduled for RARP were initially enrolled, however, 1 was excluded due to withdrawal of informed consent, 2 due to cancelled surgery, 1 due to terminated surgery, and 12 due to inconsistent body position adjustment methods with the study protocol (Figure 1). Seventy-two patients already had elevated IOP before angle adjustment; after exclusion of these participants, 135 patients were included for final analysis. The detailed IOP information was shown in Supplementary Table S2.

### Identification of angular thresholds for IOP elevation

**Figure 2:**
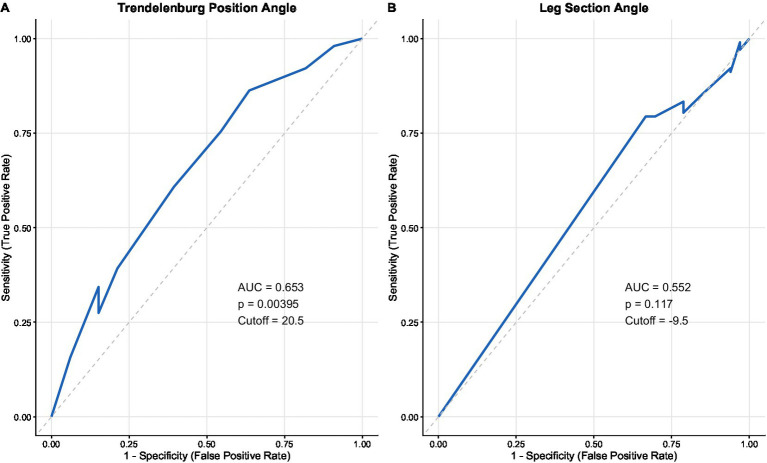
The ROC curves and best cutoff values for TREND and LEG. **(A)** The ROC curves and best cutoff values for TREND. **(B)** The ROC curves and best cutoff values for LEG.

The results revealed that for TREND, the AUC was 0.653 and the cutoff value was 20.5°, while for LEG, the AUC was 0.552 and the cutoff value was 9.5° (Figure 2 and Table 1). Based on whether the TREND angle was ≥ 20.5°, patients were divided into two groups, and baseline characteristics were compared between the groups. Compared to patients in the TREND <20.5°, patients with TREND ≥20.5° [24.61 (22.76, 26.45) vs. 23.44 (21.80, 24.42), *p* = 0.005] showed that BMI was significantly higher, while age, MAP, hypertension history, diabetes history, ethnicity, and LEG <9.5° or LEG ≥9.5° showed no significant difference between patients of the two groups (Table 2). To assess the stability of the cutoff values and their association strength, internal validation using bootstrapping showed that the AUCs for the Trendelenburg position angle group and the leg position angle group were 0.653 and 0.552, respectively.

**Table 1:** The Youden index values corresponding to different cutoff values.

Variable	Cutoff value	Sensitivity	1-Specificity	Youden index
Trendelenburg position angle				
	17.0	1.000	1.000	0.000
	18.5	0.980	0.909	0.071
	19.5	0.922	0.818	0.104
	20.5	0.863	0.636	0.227
	21.5	0.755	0.545	0.210
	22.5	0.608	0.394	0.214
	23.5	0.392	0.212	0.180
	24.5	0.343	0.152	0.191
	25.5	0.275	0.152	0.123
	26.5	0.157	0.061	0.096
	27.5	0.078	0.030	0.048
	29.0	0.000	0.000	0.000
Leg section angle				
	−29.0	1.000	1.000	0.000
	−27.5	0.990	0.970	0.020
	−26.5	0.971	0.970	0.001
	−25.0	0.922	0.939	−0.017
	−23.5	0.912	0.939	−0.027
	−22.5	0.853	0.848	0.005
	−21.5	0.833	0.788	0.045
	−20.5	0.804	0.788	0.016
	−19.5	0.794	0.697	0.097
	9.5	0.794	0.667	0.127
	1.0	0.000	0.000	0.000

**Table 2:** The basic characteristics of the two groups (*N* = 135).

Variable	Trendelenburg position angle		*p*-value
	<20.5° (*n* = 26)	≥20.5°(*n* = 109)	
Age, years	65.00 (58.00, 73.00)	67.00 (60.00, 71.00)	0.975^a^
BMI, kg/m^2^	23.44 (21.80, 24.42)	24.61 (22.76, 26.45)	0.005^a^
MAP, mmHg	95.16 (87.67, 108.08)	100.33 (91.67, 109.33)	0.231^a^
Pneumoperitoneum duration	107.50 (80.75, 124.75)	99.00 (80.00, 116.00)	0.980^a^
T12 chemosis left			0.165^b^
No	2 (7.69)	24 (22.02)	
Yes	24 (92.31)	85 (77.98)	
T12 chemosis right			0.502^b^
No	4 (15.38)	26 (23.85)	
Yes	22 (84.62)	83 (76.15)	
T13 chemosis left			0.671^b^
No	18 (69.23)	68 (62.39)	
Yes	8 (30.77)	*n* (37.61)	
T13 chemosis right			0.671^b^
No	18 (69.23)	68 (62.39)	
Yes	8 (30.76)	41 (37.61)	
Hypertension history, *n* (%)			0.377^b^
No	21 (80.77)	76 (69.72)	
Yes	5 (19.23)	33 (30.28)	
Diabetes history, *n* (%)			0.122^c^
No	26 (100.00)	97 (88.99)	
Yes	0 (0.00)	12 (11.01)	
Ethnicity, *n* (%)			>0.999^c^
Han	26 (100.00)	107 (98.17)	
Other	0 (0.00)	2 (1.83)	
Leg section angle group, *n* (%)			0.734^b^
<9.5°	5 (19.23)	27 (24.77)	
≥9.5°	21 (80.77)	82 (75.23)	

BMI, body mass index; MAP, mean arterial pressure.

a Used Wilcoxon rank-sum test.

b Used Pearson’s chi-squared test.

c Used Fisher’s exact test.

### Risk factors associated with elevated IOP

**Figure 3:**
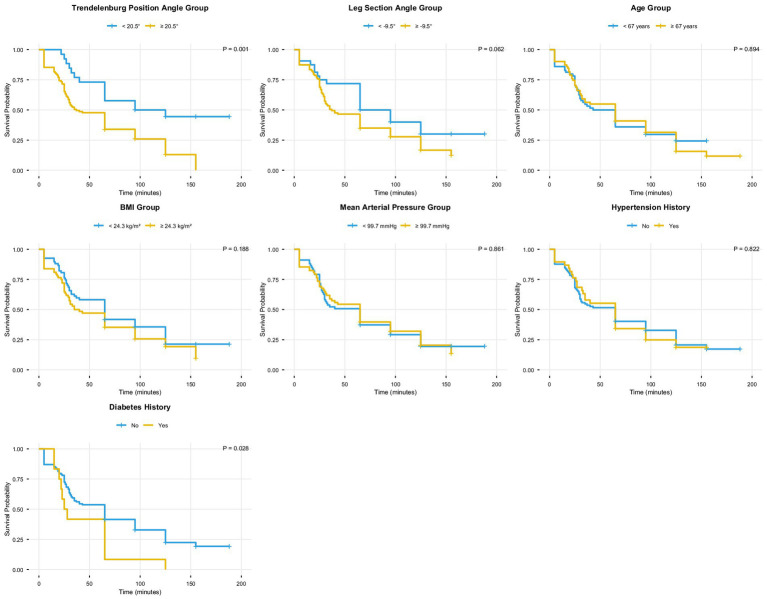
The Kaplan–Meier curves.

**Figure 4:**
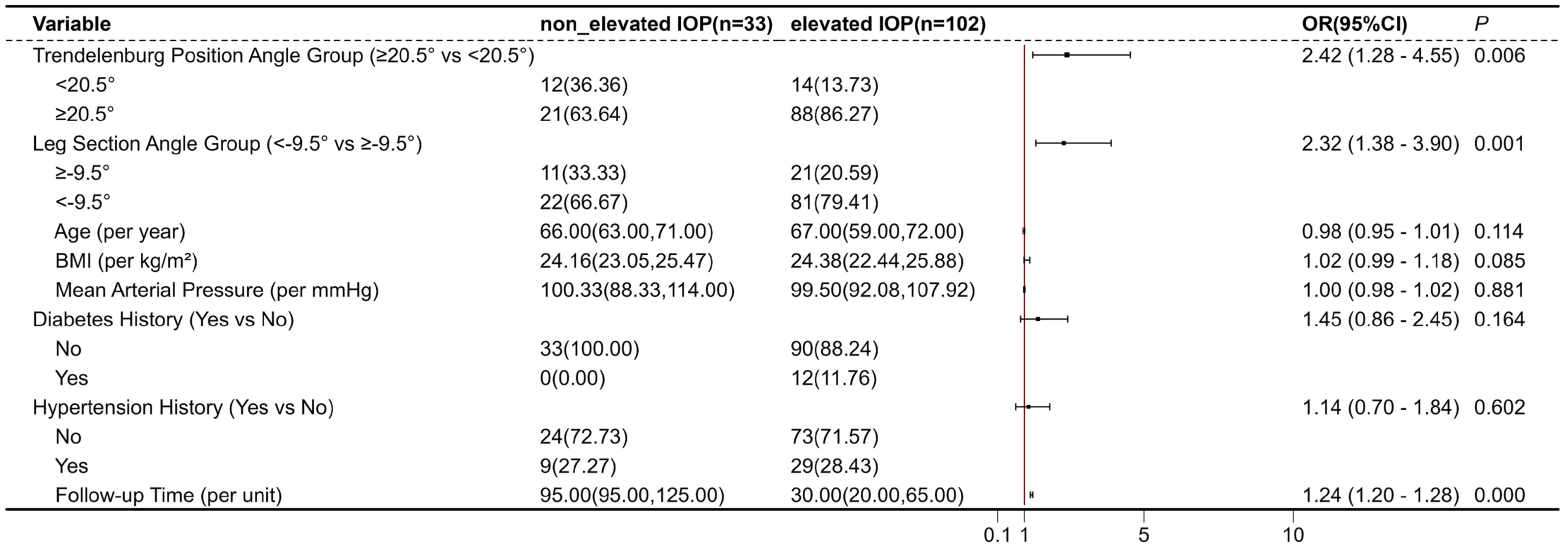
The forest plot of the generalized estimating equations results.

The univariate analysis of TREND, LEG, age, BMI, MAP, hypertension history, and diabetes history revealed that TREND (*p* = 0.001) and diabetes history (*p* = 0.028) were significantly associated with elevated (102 patients) IOP (both *p* < 0.05). Specifically, a Trendelenburg position angle ≥20.5° and a history of diabetes were identified as risk factors for elevated IOP (Figure 3). Moreover, the GEE analysis also revealed that Trendelenburg position angle group, leg section angle group, and follow-up time were significantly associated with elevated intraocular pressure (IOP) (all *p* < 0.05), indicating that a Trendelenburg angle ≥20.5°, a leg section angle ≥−9.5°, and longer follow-up time were risk factors for IOP elevation (Figure 4).

**Figure 5:**
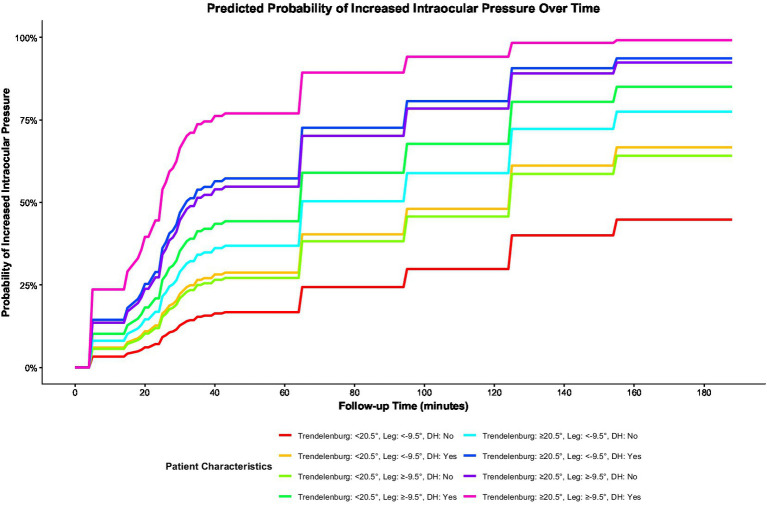
The probability of elevated intraocular pressure (IOP) occurring in different groups at different times.

To explore the risk of IOP in different subgroups, patients were classified and analyzed according to TREND and LEG threshold and diabetes history. The KM analysis results revealed that patients with a TREND ≥20.5°, LEG ≥9.5°, and a history of diabetes had the highest probability of developing elevated IOP, while those with a Trendelenburg angle <20.5°, leg section angle <9.5°, and no history of diabetes had the lowest risk (Figure 5).

### Prediction model for elevated IOP

**Figure 6:**
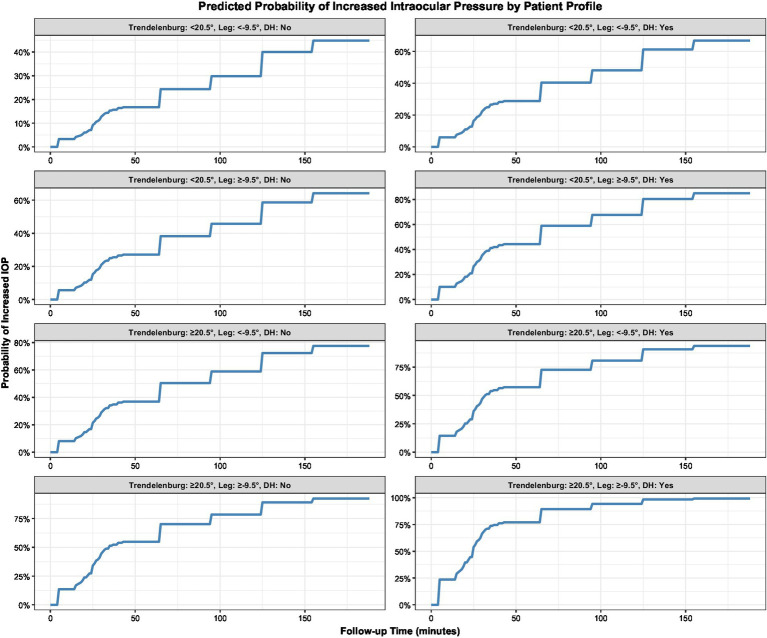
The ROC curves of the prediction model.

Based on clinical experience, TREND group, LEG group, MAP, and diabetes history were included in a binary logistic regression model to predict the occurrence of elevated IOP. The ROC curve showed an AUC of 0.721 (95% CI: 0.621–0.820) (Figure 6). A sensitivity analysis was additionally performed for the leg section angle group. After exclusion of the leg section angle group, the model performance decreased from 0.721 to 0.692.

## Discussion

This study identified several factors influencing intraocular pressure (IOP) elevation during RARP Trendelenburg positioning. The GEE analysis revealed that Trendelenburg position angle group, leg section angle group were significantly associated with elevated IOP. These results provide actionable thresholds to optimize positioning and reduce surgery-related IOP.

Although RARP is widely applied in surgeries, the Trendelenburg position is required and will lead to increased IOP. Under a 45° Trendelenburg position, Blecha et al. (12) confirmed a time-dependent increase in IOP, while a reverse Trendelenburg is a safer technique for lowering central venous pressure without significantly decreasing the systolic blood pressure in patients received hepatectomy (16).

Additionally, patient-specific factors like BMI also influence both surgical ergonomics and physiological stress. In the present study, BMI in patients of TREND ≥20.5° group was higher, however, the multivariate regression analysis showed BMI was not an independent risk factor, which indicated that BMI was a confounding factor compared to steep TREND, and the underlying reason could be partially explained by the association between higher BMI and steeper tilt requirements and longer surgical time, compounding IOP elevation (17). For example, Uchida et al. (18) identified that higher BMI and narrower pelvic dimensions were significantly associated with longer operative time. The study results also confirmed that in patients with a TREND ≥20.5°, the BMI was significantly higher than in patients with a TREND <20.5°. This suggests that in clinical practice, a steeper TREND is often required in patients with higher BMI. The forward tilt of the robotic arms also necessitates a greater degree of head-down tilt. Moreover, an increase in surgical duration and BMI could lead to increased IOP. However, the steeper the Trendelenburg angle, the greater the adverse impact on the patient’s physiological parameters during surgery. However, it should be noted that the identified threshold of 20.5° should not be interpreted as an absolute upper limit prohibiting steeper angles during surgery. Instead, our findings position this value as a critical risk warning threshold. The clinical utility of this finding lies in fostering an individualized positioning strategy rather than a rigid protocol. It is also worth acknowledging a notable discrepancy between our statistical findings and clinical reality regarding patient positioning during RARP. While our analysis identified 20.5° as the optimal threshold for TREND to minimize IOP elevation, clinical practice often necessitates steeper angles, particularly in patients with higher BMI who require greater tilting to achieve adequate pelvic access. We recognize this as a limitation of the present study. Future studies should focus more on higher BMI patients and define an individualized threshold for them.

For patients with pre-existing risk factors—such as a history of diabetes, glaucoma suspect status, or advanced age—this threshold serves as an early alert for anesthesiologists and surgeons to exercise heightened caution. It encourages the surgical team to adopt a “lowest effective angle” principle: while adequate exposure is paramount for robotic dexterity and oncological safety, the angle should be titrated to the minimum necessary to achieve the surgical field, particularly in high-risk cohorts. By recognizing 20.5° as a inflection point for risk stratification, clinicians can make more informed, real-time decisions to balance surgical ergonomics with ocular safety, potentially mitigating the risk of postoperative visual complications without compromising procedural success.

Apart from BMI, operative time, and other indices, the present study found TREND and LEG were also significant risk factors for increased IOP during surgery, a previous study performed by Khan et al. (19) revealed that when TREND was 30°, intra-abdominal insufflation pressure and systolic blood pressure are two factors that significantly associated with IOP during RARP; while Balkan et al. (20) revealed that a significant increase in IOP was found during robot-assisted laparoscopic prostatectomy under a steep Trendelenburg position (35° to 45°), the study performed by Ristin et al. (21) also confirmed that steep Trendelenburg positioning causes an increase in IOP only after 1 min of Trendelenburg position. While in the present study, the TREND threshold was 20.5°, although compared to previous studies, the angle was relatively small, it remained a significant risk factor for increased IOP. Head position was another important influencing factor for IOP. Raz et al. (22) reported that a modified Z-Trendelenburg position (head elevation 5°–10°) significantly reduced intraoperative IOP; however, the interplay between Trendelenburg angle, head elevation, and surgical duration remains poorly quantified. In the present study, the Trendelenburg angle cutoff was 20.5°, and the LEG cutoff was 9.5°, providing clinically relevant references for surgical positioning. These findings suggest that to avoid increased IOP during surgery, a suitable position strategy should be employed.

A comprehensive elevated IOP prediction model based on the three factors (Trendelenburg angle, leg angle, and diabetes history) was also developed in the present study, compared previous studies, Fan et al. (23) proposed an elevated IOP prediction model based on clinical features and achieved the accuracy of 0.7944, while Ida et al. (24) revealed that pseudoexfoliation syndrome and preexisting glaucoma were risk factors for increased IOP after surgery. However, we did not find any study focused on the prediction of elevated IOP based on the position angles and clinical features, and the results in this study achieved an AUC of 0.721, which indicated moderate performance. Therefore, a more accurate and personalized prediction model should be further proposed in future studies.

However, this study still has several limitations. First, although we identified several cutoff values, some features (BMI and surgical duration) are difficult to control in practice. For example, it is not feasible to reduce a patient’s BMI to ideal levels within a short period, and the surgical duration is often unpredictable. Secondly, partly due to the small sample size, the diagnostic performance of the proposed model was suboptimal, and a future large-scale multicenter study should be performed to validate our results. Finally, although we revealed that diabetes was an independent risk factor, details including disease duration, glycemic control, or the presence of diabetic retinopathy were not recorded, some other parameters (PaCO₂, peak airway pressure, central venous pressure) were also not recorded, in the future prospective study, we will include these parameters for analysis.

This study demonstrates that a Trendelenburg angle ≥20.5°, leg angle ≥9.5°, and a history significantly elevate IOP during RARP. The identified thresholds provide actionable insights for optimizing patient positioning to mitigate IOP-related risks, such as postoperative vision loss. While anesthesia initially counteracts IOP elevation, prolonged steep positioning and metabolic factors exacerbate ocular hypertension. These findings underscore the importance of individualized positioning strategies and further research to refine safety protocols in RARP. Clinicians should consider these thresholds to balance surgical exposure with patient safety, particularly in high-risk populations.

## Data Availability

The original contributions presented in the study are included in the article/Supplementary material, further inquiries can be directed to the corresponding authors.

## Supplementary material

SUPPLEMENTARY FIGURE S1 Trendelenburg position.
