# Supplementary figures and images for: Angular threshold for intraocular pressure elevation during robot-assisted radical prostatectomy in Trendelenburg position
Source: Front Med (Lausanne). 2026 May 15;13:1769460. doi: 10.3389/fmed.2026.1769460 (PMC13219256; doi:10.3389/fmed.2026.1769460)

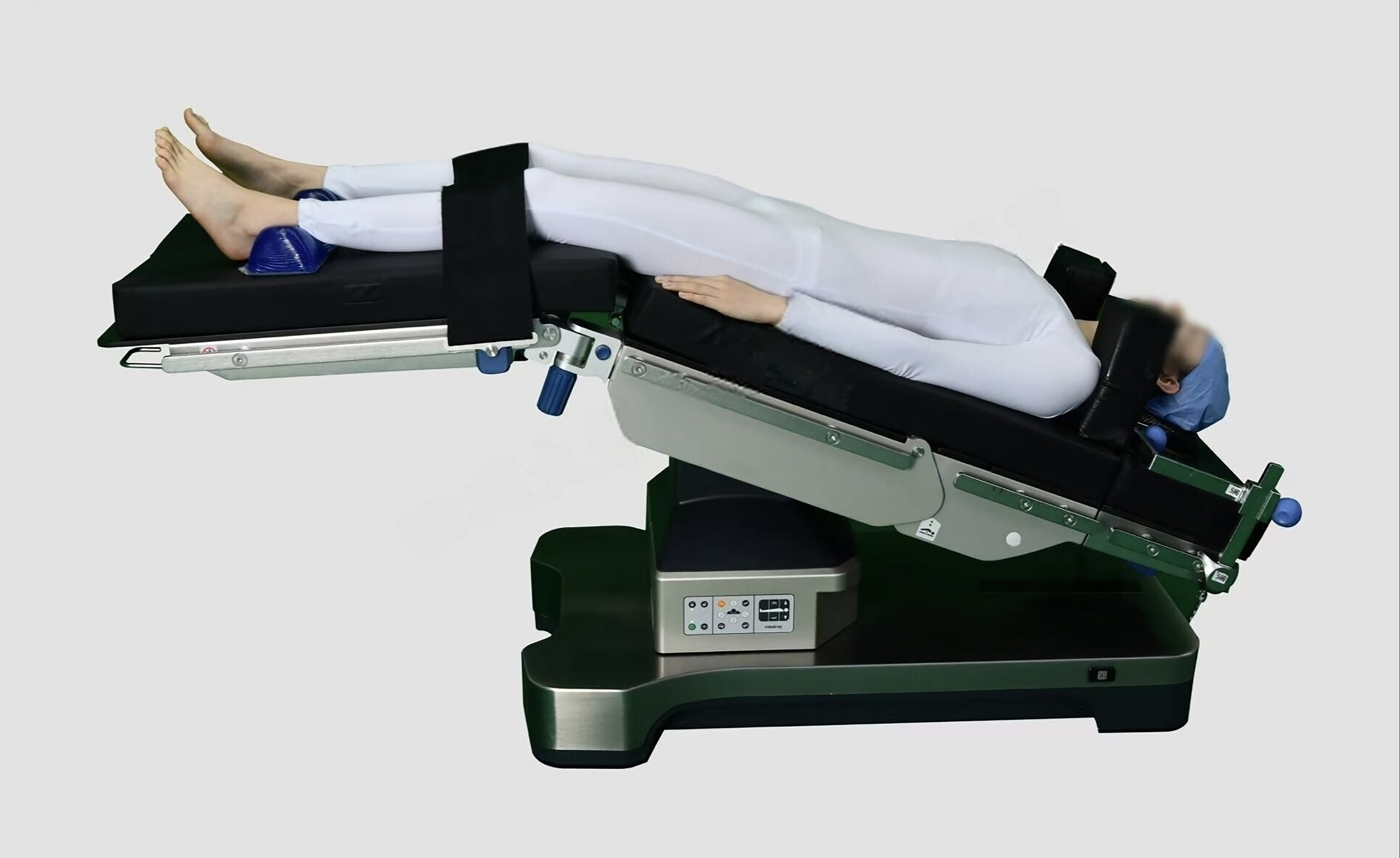

Supplement: SUPPLEMENTARY FIGURE S1 — Trendelenburg position. [file Image_1.jpeg]
