# Supplementary material for: Angular threshold for intraocular pressure elevation during robot-assisted radical prostatectomy in Trendelenburg position
Source: Front Med (Lausanne). 2026 May 15;13:1769460. doi: 10.3389/fmed.2026.1769460 (PMC13219256; doi:10.3389/fmed.2026.1769460)
Supplement: Supplementary file 2 [file Table_1.docx]

**Table S1.** Time points of Intraocular Pressure and Chemosis measurements

| Time points of measurements | | Intraocular Pressure | Chemosis |
| --- | --- | --- | --- |
| T1 | Supine position before induction of anesthesia | X | - |
| T2 | Supine position after induction of anesthesia | X | - |
| T3 | Trendelenburg Position for 5 minutes | X | - |
| T4 | Maintain the Trendelenburg position and establish pneumoperitoneum | X | - |
| T5 | Head-down tilt for 1 hour | X | - |
| T6 | Head-down tilt for 1.5 hours | X | - |
| T7 | Head-down tilt for 2 hours | X | - |
| T8 | Head-down tilt for 2.5 hours | X | - |
| T9 | Head-down tilt for 3 hours | X | - |
| T10 | Maintain the Trendelenburg position and release of the pneumoperitoneum | X | - |
| T11 | Return to the supine position but maintain anesthesia | X | - |
| T12 | After awakening from anesthesia | - | X |
| T13 | 24 hours after surgery | - | X |

T1: Supine position in the awake state
T2 and T11: Supine positions before and after Trendelenburg positioning under anesthesia, respectively
T4 and T10: At the time of pneumoperitoneum establishment and release, respectively, in the Trendelenburg position
T5 to T9: Measurements taken every 30 minutes during the first 3 hours in the Trendelenburg position
