# Supplementary material for: Angular threshold for intraocular pressure elevation during robot-assisted radical prostatectomy in Trendelenburg position
Source: Front Med (Lausanne). 2026 May 15;13:1769460. doi: 10.3389/fmed.2026.1769460 (PMC13219256; doi:10.3389/fmed.2026.1769460)
Supplement: Supplementary file 3 [file Data_Sheet_2.pdf]

Table 2 Mean Intraocular Pressure of Left and Right Eyes at 11 Measurement Points

Point	IOP（Left）	IOP（Right）
1	16 (13, 20)	16 (13, 20)
2	14 (12, 15)	14 (13, 15)
3	17 (15, 21)	17 (15, 21)
4	22 (18.5, 26)	23 (18, 27)
5	23 (20, 26)	23 (19, 27)
6	24 (20, 26)	23 (19, 28)
7	25 (22, 28)	26 (22, 28)
8	25.5 (21, 28)	26.5 (23, 28)
9	28.5 (25.75, 30.25)	28 (25, 30)
10	24 (20, 27)	24 (20, 27)
11	18 (15, 22)	18 (15, 22)
